# Evaluation of the consultation program in Shiraz University of Medical Sciences

**Published:** 2014-01

**Authors:** RITA REZAEE, PARISA NABEIEI, MOHAMMAD MAHDI SAGHEB

**Affiliations:** Quality improvement in Clinical Education Research Center, Shiraz University of Medical Sciences, Shiraz, Iran

**Keywords:** Advisor, Consulting, Guidance, Education

## Abstract

**Introduction**: Consultation and guidance is a process of learning which is done through the relationship between two individuals. In this mutual relationship, the ounsellor, through his scientific and occupational skills and qualification, tries to help the students using the methods corresponding to their needs. The main objective of this study was to provide a framework for the management of the advisors’ plans in the university based on the analysis of different schools.

**Methods:** This research is a cross-sectional and descriptive-analytic study. Data were collected both qualitatively (centralized groups at the presence of advisors in universities) and quantitatively (self-assessment of teachers and students' evaluation). Sampling was done randomly from all students of Shiraz University of Medical Sciences. All the teachers who were advisor took part in this study.

**Results**: This study was conducted in eight schools of Shiraz University of Medical Sciences simultaneously and 974 students and 125 teachers took part in it. At the time of data collection, 25.5 percent of the students declared that the advisors have provided allocated time to them and 45.4 percent believed that the advisors helped the students to understand the importance of the courses.

**Conclusion: **The emergence of weaknesses and strengths of the academic advising program and the guidance through teacher's self assessment, not only may be the sign of realization of programmed objectives, but also may be the starting point for qualitative improvement of the situation. Moreover, in order to improve the advisory services and guidance, it is necessary to regard consultation as a scientific subject which needs training.

## Introduction

Consultation and guidance is a process of learning which is done through the relationship between two individuals. In this mutual relationship, the counselor, by his scientific and occupational skills and qualifications, tries to help the students with the methods corresponding to their needs (1).

Consulting services have been offered to students non-formally and very limited by the school members since the advent of the formation of high education centers. However, student services, including advice by experts appeared between the First World War and the economic downturn of the twentieth century (1930) and grew rapidly in higher education. These services became common by Williamson, the student deputy of Minnesota University of the United States of America, since the seventh decade of the twentieth century (1970) it has been regarded as a part of student affairs in universities and higher education institutions of America. Consulting services, either scientific or specialized, began in Iran by the foundation of the first consultation centers which were first founded in Tehran and then in other universities gradually. In (1994), the regulations for the foundation of culture and higher education to the affiliated universities was considered in student deputies' congresses and now the consultation centers are inaugurated in 93 universities and affiliated higher education institutions of Ministry of Science, Research and Technology and also Health, Treatment and Medical Education and some faculties of Azad University (2).

According to the Iran Consulting Committee, consultation is a process which is based on a helpful, face to face, and specialized relationship through which the counselors, using their knowledge and special skills, pave the way for the growth and problem solving of their visitors (3).

As the society is so expanded and the relationship between individuals is complex, the need for providing consulting and guiding services is more tangible and the individuals who are often faced with changes of life situations will experience psychological pressure or excitement either positively or negatively. Obviously entering the university, as a new experience besides other issues such as new educational conditions, different interpersonal relations, new and unfamiliar living environment, family leaves and …, affects the student's compatibility (4).

In Shiraz University of Medical Sciences in coordination with other universities and aiming at objectively guiding the students’ affairs for their better growth and flourishing  by setting objectives, duties and clear executive framework for preventing their educational failure, paving the way for the advancement of scientific background and solving educational, research, individual, social and welfare problems, the central advisor committee has been established in (2006) by the president of the department of education who had the responsibility for executing regulations of advisors. This committee has been formed on the basis of regulations of ministry and the order of the university president and its domain of action is compatible to the latest result of advisors regulation of the supreme council of programming of medical sciences (5).

Providing a good educational training consultation is an indicator which is often ignored in the duties of a college (6). In recent decades, the evaluation of the higher education institution's functioning has been regarded as an important yardstick in determining the quality of the action of the organization. So, any kind of study related to the educational progress of the students can be considered as a step for growth and advancement of the quality of educational institutions, specially the universities. Obviously, the advisor plan will achieve its goals in the universities

The importance of consulting and evaluation of consulting programs have been emphasized in recent investigations, showing that only 29% of university institutions evaluate the effectiveness of advisors’ activities (7). The evaluation of the effectiveness of advisors shows the importance of consultation as a professional responsibility of teachers. Individuals tend to be involved more in a kind of job which is evaluated. The job which is not evaluated would have a downward trend. Moreover the content of an evaluation tool is not only for measuring its reality, but also it can be used as an indicator of desired behavior and a motivation for improvement of the ideal professional behavior (what that should be).

The quality of evaluation of academic consultation program, often based on the consequences of the program, is viewed as the primary indication of success or failure. This success or failure is affected by other factors which are often ignored. The council for advancement of standards in higher education has set 13 standards for educational consultation program including. 1. Mission, 2. Program, 3. Leadership, 4. Organization, 5. Human resources, 6. Financial resources, 7. (facilities, technology, equipment), 8. Familiarity with legal responsibilities , 9. Providing equal opportunities, 10. Communication establishment, 11. Diversity, 12. Ethics observation, 13. Assessment (8).

A great number of studies have been conducted on different aspects of the educational consultation programs in universities. For example, in the results of Cusee's study in 2002, it was indicated that students would prefer advisors who are accessible, have enough knowledge and skill, are easy to speak with and dependable (9).

Also the results of another study done by Rajaee et al. in 2004 show that the students who have benefited from educational consultation have more educational progress and this is the result of the increase in educational skills and on the other hand the students’ participation in group sessions and discussions which result in their higher self-confidence and more activities (10).

In another study done in 2001 by Lowa and Toney, it was found that students have different views on their understanding about the importance of consultation responsibilities according to their situations, and it was recommended that experienced advisors should be trained in universities; their responsibilities should be defined while they are provided with facilities for their access, ability for response, assessment and reward (11).

Since the application of advisor plan is important in Medical Sciences Universities, the recognition of the present situation and assessment of this plan would pave the way for the advancement of this situation. The verbal polling from different individuals involved in this process made it clear that there were different shortcomings, problems. Therefore, this study was conducted aiming at of surveying the present situation and assessing all aspects of this plan.

## Methods

This study was done to analyze the functioning of advisor plan in Medical Sciences Universities.  Data were collected from teachers and students as to the services provided in order to find out the effectiveness of the plan. 

This study was conducted not only for assessment of the effectiveness of the plan, but it was also based on the process of its implication. So data collection was performed both qualitatively (centralized groups in presence of advisors in universities) and quantitatively (self-assessment of teachers and students' evaluation).      

After the groups were decided, the teachers of each school were asked to express their problems and suggestions in the form of brainstorming. At the end of each session, the responses were categorized, unified and summarized by the members. In the next stage, the questionnaire was made and its validity was confirmed by experts. Cronbach's coefficient was used to determine its reliability which was found to be 83%. Tee second questionnaire was designed to evaluate the consulting teachers.

This questionnaire consisted of two parts, the first part including 3 questions about demographic specification (sex, field of study and experience); the second part included 9 open questions on the weak and strong points about the consultants. The self-evaluating questions of the questionnaire in different faculties included consultant teachers, attitude to consultation process, understanding the students, attitude about the consultation process. Suggestions for improving the program and expression of 3 good characteristics in teachers' point of view were discussed as strengths and weakness. It is noteworthy that content validity of this questionnaire was confirmed by experts and reliability was obtained 81% by using Cranach's coefficient. Data analysis was done by SPSS 14 (SPSS Inc, Chicago, IL, USA) software. 

## Results

This research was conducted in eight schools of Shiraz University of Medical Sciences on 974 students. Among them, 324(32.9%) were men and 650(67.1%) were women. Table 1 indicates distribution of the teachers and students in each schools.

**Table 1:** Students' and teachers’ frequency distribution in different schools

**Schools**	**Students**	**Teachers**
	**Frequency (%)**	**Frequency (%)**
**Medical**	66 (6.8)	24 (19.2)
**Paramedical**	37 (3.8)	6 (4.8)
**Nursing**	258 (26.4)	32 (25.6)
**Rehabitation**	78 (8)	11 (6.8)
**Health**	107 (11)	19 (15.2)
**Pharmacy**	142 (14.6)	24 (19.2)
**Dentistry**	210 (21.6)	9 (7.2)
**Management**	76 (7.8)	24 (19.2)

Table 2 indicates students’ perception about teachers consulting in Shiraz University of Medical Sciences.

**Table 2:** Students’ perception about teachers’ consulting

**No.**	**Questions**	**Disagree (%)**	**No comments (%)**	**Agree (%)**
**1**	Teacher allocates enough time to students	26.8	20.7	52.5
**2**	Teacher encourages students to get help	35.1	21.7	43.2
**3**	Teacher encourages students to express their feeling	30. 5	22.2	47.3
**4**	Teacher is a good listener	22.1	19.7	58.2
**5**	Teacher gives the students the correct information	22.3	22.5	55.1
**6**	Teacher help the students understand the importance of the lessons	30.7	24	45.4
**7**	Teacher pays attention to students as a person	33.3	23.9	42.8
**8**	Teacher helps the students with long-term educational programming	40	24.4	35.6
**9**	Teacher provides relationship with other supportive and commutative resources	43.6	26.3	30.2

At the end of the strengths and weaknesses points of this program, according to the teachers’ comments were mentioned at table 3.

**Table 3:** Strengths and weaknesses points of the consulting program

**Schools**	**Strengths**	**Weakness**	**Suggestion**
**Medical**	Establish supervising center	Inadequate funding	Introduce talented students to the talented students office
	Insufficient number of counselors	Lack of referring of some students to the advisor	Advisors’ contact with students’ families
	Evaluation of the advisor at the end of the semester and provision of feedback	-	Use of psychological tests to identify vulnerable students
	-	-	Prepare the site of consultation at university and put some information, regulations and forms on it
	-	-	Organize some expeditions for students and advisors
**Nursing**	Refer most of the students to counselors	-	Meeting with students and advisor at the beginning of the semester
	Good relationship of students with advisors	-	Workshops and educational pamphlets for students
**Dentistry**	-	Lack of basic science students at schools for this program	Regular meetings by students and advisor of the school
	-	Failure to perform annual evaluations of advisor	-
	-	Lack of interest of teachers in consulting program	-
**Rehabilitation**	-	Disproportionate allocation of male and female students with advisors	Referring students to advisors at least one time during the semester
**Heath Management and Informatics**	Counselors' evaluation at appropriate times	Low number of advisors as compared to students	Counselors' meetings at least once each semester
	-	Lack of knowledge of some instructors about how to consult students	Confidential counseling sessions
	-	Resistance of some teachers against counseling program	-
	-	Lack of interest of advisors in attending the mentoring program	-
**Para Medical Sciences**	-	Lack of counselors' time to complete and report consulting forms	Referring advisors to preparing daily reports about students
	-	Lack of experts to follow-up counseling	-

## Discussion

Accessibility of consultant teachers is one of the characteristics that impact the consultation quality and satisfaction. Similar to the result of this study, 52.5% of the students stated that they have access to their consultant teachers. Most of the university students believed that the consultant teacher allocates enough time to students. 

Legutku's study showed that the students of American universities considered the consultant teacher as an accessible person who allocates enough time to them for consultation.. Nowadays, students encounter higher social and personal pressure (12), so they look for someone out of home and in university who is confidential, alert, good listener, accessible, someone who presents solutions to the problems.

In this study, most of the students believed the consultant teachers encourage them to express their feelings and receive help. This is probably due to the fact that one of the consultant teacher's duties is to establish trust and motivation through listening to students, confirming their feelings and thoughts, accepting the students’ inspiration, disliking their particular behaviors, and understanding and respecting their culture and class differences. 

Also most of the students believe that the consultant is a good listener. Active listening, as effective communication with students, is one of the most important duties of the consultant teachers. 

Adhami's study results showed that students tended to express their feelings in the limited time the consultant allocated to them and this showed that attention to student's feelings, besides their educational affairs, could be effective in their improvement. The consultant teacher’s responsibility is to provide students with correct content (13). According to this study’s results, 55.1% of the university students stated that their consultant teacher has provided them with appropriate content.

Advisors' information and knowledge can provide proper background for providing services to students. Legato's study conducted in America in 2006 showed that students evaluate their consultant teachers as knowledgeable, understanding and accessible when needed (5).

As students don’t have an appropriate understanding of their abilities and talent and are unaware of their future job perspectives, they are regard their consultant teachers as successful models who can create positive attitudes in them by considering the importance of lessons and professional perspective.  

Superfast defines job consultation as a process of helping one to have a good image of him/her and his/her job, leading to satisfaction about himself/herself and his/her society. Research has shown that professional and employment consultation needs among the students are high. Job readiness program is one of the basic student's processional needs and about 80% of students need help and guidance in identifying job opportunities related to their fields of study. Recently, 82% of Americans have mentioned finding a good job as their reason for attending university.

In 1995, in evaluating student's psychological problems, which stated that 20% of student's psychological problems are about their job consultation needs. In this study, most of the students believed that consultant teacher helps them in educational long-term planning and their academic progress. Results of some research show that consultation affects the student's improvement in educational performance and those with better educational background are more effected (13).

Rajaee et al., in his study on 2 control and study groups, showed a significant difference between them (p=.003) (10). So, educational consultation is effective on increasing their academic achievement and this is consistent with the results of Tizro (7).

Turner et al. (2001) (14), Shams et al. (2000) (8), Adhami et al. (2008) (6) reported that most of the students believe that consultant teacher doesn't link them to other supportive and consultant resources. Adhami in his study indicated that one of the duties of the consultant teacher is introducing students to different centers in the university (6) and the students believed that the consultant teacher’s guidance for their obviating is at a low level; this is consistent with the results of this research. So it is important to consider consultation as a responsibility which needs education. In order to improve consultation and guidance, competent individuals must be used to introduce students to supportive and consulting centers.

## Conclusion

In surveying the consultant teachers' comments in this study, strengths and weaknesses of the program were assessed; they can be an indicator of achieving objectives and a means for improving the present status because all social systems should improve their quality so that they can function effectively. 

Quality is defined according to the society and addressee's needs, which they are changing all the time. So, all systems should improve their quality to match themselves with such changes. Higher education systems have a special condition. Since so many people benefit from their productions, it encounters so many needs and improving the quality in this system is crucially important. 

**Suggestions**

1. Holding seminars for consultant teachers and related committee formations.
2. Holding orientation programs for new students and holding different workshops for teachers and students.
3. Making consultation optimal for all the students.
4. Making consultant teacher a constant one until the end of school time except the medical school in which the consultant teacher must change.

**Research limitation**

The main limitation of this research is lock of comparison between the students currently under consultation with those who have refused counseling. This research is the initial step for future research in order to increase the quality of consultation.
